# Isolation of specific and biologically active peptides that bind cells from patients with acute myeloid leukemia (AML)

**DOI:** 10.1186/1756-8722-1-8

**Published:** 2008-07-10

**Authors:** Naomi Galili, Emmanuelle Devemy, Azra Raza

**Affiliations:** 1 Saint Vincent's Comprehensive Cancer Center, New York, NY, USA; 2 McGill University, Montreal, Canada

## Abstract

**Purpose:**

In a departure from conventional strategies to improve treatment outcome for myeloid malignancies, we report the isolation of leukemia-specific peptides using a phage display library screened with freshly obtained human myeloid leukemia cells.

**Results:**

A phage display library was screened by 5 rounds of biopanning with freshly isolated human AML cells. Individual colonies were randomly picked and after purification, biologic activity (growth and differentiation) on fresh AML cells was profiled. Ten peptides were synthesized for further biological studies. Multiple peptides were found to selectively bind to acute myeloid leukemia (AML) cells. The peptides bound to leukemia cells, were internalized and could induce proliferation and/or differentiation in the target patient cells. Two of the peptides, HP-A2 and HP-G7, appeared to have a novel mechanism of inducing differentiation since they did not cause G1 arrest in cycling cells even as the expression of the differentiation marker CD11b increased.

**Conclusion:**

Peptide induced differentiation of leukemia cells offers a novel treatment strategy for myeloid malignancies, whereas their ability to induce proliferation could be harnessed to make cells more sensitive to chemotherapy. Conceptually, these leukemia specific peptides can also be used to refine diagnosis, document minimal residual disease, and selectively deliver toxins to malignant cells.

## Background

We proposed to isolate leukemia specific peptides that have the potential to target and deliver toxins to acute myeloid leukemia cells (AML) or to modify the biological behavior of the cells to which they bind using a phage display library. First described by George Smith in the mid 1980s [1], this technique allows repertoires of antibodies, proteins or peptides displayed on the surface of phage particles to be screened by any chosen target. Single chain Fv phage libraries have been used to isolate antibodies that recognize cell surface antigens for clinical, diagnostic and therapeutic applications [2-6] or for antigen epitope mapping [6,7]. An alternative approach was to use peptide phage libraries to identify small molecules that can bind either purified targets or cell surface receptors. Peptides that are specific for surface expressed immunoglobulins isolated from chronic lymphocytic leukemia (CLL) cells [8] and multiple myeloma cells [9] have been identified for potential patient specific targeted therapy. Our approach was to use freshly obtained patient cells to isolate leukemia specific peptides from a phage library. In this study we show that multiple myeloid leukemia specific and non-specific peptides can be identified by this method. In addition, we show that these peptides are capable of altering the biological behavior of AML cells while having no effect on normal marrow elements.

## Methods

### Patients and normal donors

Specimens were obtained from patients with chronic myelogenous leukemia in blastic crisis (CML-BC), AML of different FAB classifications, as well as from normal donors. Informed consent was obtained from all patients prior to study according to the regulations of the Institutional Review Board of Rush Medical Center.

### Cell preparation

The peripheral blood (PB) or bone marrow (BM) cells were subjected to density cut centrifugation over Ficoll-Hypaque. The mononuclear fractions were washed twice and used directly. Granulocytes were obtained from the high-density fraction. Immature CD34+ stem cells were isolated by magnetic cell sorting and separation (Miltenyi Biotec, California). The promyelocytic cell line HL60 (ATCC) was maintained in RPMI 1640/20% FBS. Cells were induced to differentiate into the granulocytic lineage by treatment with 1.5% (v/v) of DMSO during 7 days of culture.

### Isolation of phage binding to human malignant myeloid cells

This work was performed using the Display PHAGE system library, purchased from Display System Biotech (Vista, California) with a diversity of approximately 3 × 10^7^.

Initial selection of phage specific for myeloid leukemia used cells from patients with CML-BC. CML-BC cells were incubated with the library (10^12 ^cfu) in PBS/0.1% milk at 4°C for 90 minutes. Unbound phage was removed by 5 washes with buffer. Weakly bound phage was acid eluted by glycine (0.1 M, pH2.2) followed by cell lysis (30 mM Tris pH8.0, 1 mM EDTA) to elute the tightly bound phage. This second fraction was amplified by re-infection and growth in E. Coli. Amplified phage was purified by PEG precipitation and used for another round of binding. The "biopanning" step was repeated five times to obtain a population that was highly enriched with phage expressing peptides that bind to leukemia cells. After growth on agar plates using antibiotic selection, individual colonies were randomly picked, amplified, and PEG purified. Binding to leukemia cells was confirmed by ELISA assay.

### ELISA Assay

Amplified phage from a single colony (10^9^cfu/ml) were dispensed into a 96-well plate containing either leukemia or normal peripheral or bone marrow mononuclear cells (250,000 cells/well) previously fixed with glutaraldehyde and blocked with PBS/2% milk. After 2 hours, wells were washed and bound phage was detected by a monoclonal anti-M13 antibody HRP conjugate (1:2,000 in PBS/1% milk, Amersham Pharmacia Biotech, NJ). Following addition of HRP substrate, intensity of color was measured by spectrophotometry. Each phage clone was tested in triplicate with appropriate controls. Identical assays were performed using synthesized peptides that had been conjugated with biotin. The bound peptide was detected by streptavidin-HRP diluted 1:1,000 in PBS/1% milk (Amersham Pharmacia Biotech, NJ). A biotin control confirmed that binding to the cells was via the peptide moiety itself and not via the biotin conjugate.

### Peptide synthesis

Phage DNA was extracted using the QIAprep M13 kit (Qiagen). The hypervariable oligonucleotide sequence coding for the peptide was PCR amplified using the following primer set: Primer 1 = 5' GGG ATT TTG CTA AAC AAC 3', Primer 2 = 5' GGA GGT CTA GAT AAC GAG 3'. Each clone was amplified, purified and sequenced (Gene Link, NY) in duplicate. The synthetic octapeptides with terminal cysteine residues were commercially synthesized (New England Peptide, Inc., Massachusetts) with more than 95% purity. Peptides were not cyclized by oxidation; therefore percentage of free sulfhydryl groups was evaluated by Ellman's reaction using a cysteine standard (Pierce Biotechnology). This reaction allowed us to determine the percentage of reduced cysteines in the peptide solution used for our experiments. The percentage ranged between 50% and 75% for most peptides except for HP-B6 (100%) and HP-G2 (30%). A biotin molecule was conjugated to the N-terminal of the peptide for cytochemistry studies.

### Cytochemistry

Fresh cells, blocked with PBS/4% BSA for one hour at 37°C, were incubated with biotin-conjugated peptide at 1 μM for 30 minutes unless otherwise stated. Free biotin was used to confirm that binding of biotin-conjugated peptide was mediated by the peptide moiety. After washing, cells were fixed with 3.7% paraformaldehyde, permeabilized with methanol and incubated with streptavidin-FITC (DAKO) diluted 1:500 in PBS/4% BSA for 30 minutes at room temperature and washed again. Alternatively, cytospin preparations of cells were fixed in 3.7% paraformaldehyde and kept at -80°C for further study. The fluorescent signal was analyzed using AxioVision 2.05 software.

### Liquid culture of HL60, AML and normal cells

To assess the biological effects of peptides on growth and differentiation of HL60 cells, logarithmic growth phase cells were seeded in 3 ml of RPMI 1640/10% FBS. Cultures in the presence or absence of a single peptide (10^-6 ^M -10^-4 ^M) or DMSO (1.5%) were assessed for the level of cell viability, cell number, and differentiation, after 7 days without changing the medium.

AML or normal bone marrow cells were seeded at a concentration of 10^6^cells/ml in 3 ml of RPMI 1640/15% FBS. Peptides were added at a single dose of 10^-4 ^M without addition of fresh medium or peptide for the duration of the cultures. Granulocyte-Macrophage Colony-Stimulating Factor (GM-CSF, 100 U/ml) was used as a positive control. Viability was evaluated at day 14.

### Analysis of cell cycle

After 7 days of culture, HL60 cells (1 × 10^6^) were washed in PBS, fixed in 1% paraformaldehyde and permeabilized with 0.5% Triton X100 in acid solution. After neutralization, cells were incubated with RNase A and stained with propidium iodide. The relative DNA content was analyzed by flow cytometry.

### CD11b analysis by flowcytometry

After 7 days of culture, HL60 cells (1 × 10^6^) were stained with fluorescence conjugated monoclonal antibodies recognizing the myeloid maturation marker CD11b (Becton Dickinson Immunocytometry system) for 20 minutes at 4°C. The percentage of cells with a fluorescence intensity above the control was measured.

### Colony assay

Mononuclear cells (10^4 ^cells/ml) were plated in 0.8% methylcellulose (Methocult, StemCell Technologies Inc., Vancouver, Canada) in Iscoves modified Dulbecco medium (IMDM, Gibco Laboratories, Grand Island, NY) with the peptides at a single dose of 10^-4 ^M or GM-CSF at 100 U/ml without addition of fresh medium or peptide. The solvent used for peptide preparation served as a control. Following 14 days of incubation, the number of colonies was assessed and cytospin preparations were made for Giemsa morphology staining. Percentage of differentiated cells was evaluated by counting 200 cells.

### Statistical analysis

All analyses were performed using SYSTAT software version 10.0. The relationship between AML patients' clinical data and responses of AML cells to the peptides was analyzed by Fisher's exact test.

## Results

### Isolation of phage able to bind cells from leukemia patients

Phage clones that bind to leukemia cells were isolated by incubating a library of M13 phage bearing 8 mer peptides with BM mononuclear cells from CML-BC patients. After five rounds of biopanning, 450 individual phage were picked randomly and tested by ELISA against cells from CML-BC patients to confirm the phage binding. Only clones with an O.D at least two times higher than controls were considered positive for the specimen studied and only those positives for at least three different CML-BC cell populations were considered leukemia positive. Among the 450 clones, 129 (28%) were positive for 3 different CML-BC patients. These clones were then profiled with AML and normal cells. Sixty-eight (15%) clones bound to CML-BC and AML cells, and were used for further studies with PB and BM cells from normal donors. Of these 68 clones, 18 did not bind normal PB specimens (n = 3), and 17/18 did not bind normal BM specimens (n = 3). The 68 phage clones were sequenced and 51 gave a good sequence chromatogram. Twelve different sequences of 8 amino acids surrounded by two cysteine residues were detected with an enrichment of the 3 sequences: CVSEDIYDAC (22/51), CEFQQWSGKC (8/51) and CNHVCSRLGC (7/51). Table 1 shows the sequences as well as the binding characteristics of the phage tested on leukemia and normal cells. Two phage, HP-G2 and HP-C8, differ by two amino acids and displayed different binding profiles. To confirm the specificity of the clones for leukemia cells, 4 clones were tested on isolated normal marrow CD34+ cells (under-represented in normal donors). No binding was detected. The binding profile of the phage clones was nearly identical for AML and CML-BC cells, implying that the binding of the peptides was myeloid leukemia specific rather than CML-BC specific. Since AML specimens were more readily available, all further studies were done using AML cells.

**Table 1:** Peptide sequences and binding profiles of phage tested on leukemia and normal cells in an ELISA assay.

Phage Clones	Sequences	AML BM	CML BM	Normal PB	Normal BM	Normal CD 34+
HP^A^-A6	CNETTVREYC (4)	+	+	ND^B^	ND	ND
HP-A2	CIEETARKGC (2)	+	+	-	-	-
HP-B6	CNHVCSRLGC (7)	+	+	-	-	-
HP-G7	CNELHMKQHC (2)	+	+	-	-	-
HP-G2	CNNATFEDGC (1)	+	+	+	-	ND
HP-C8	CNNATVEDEC (1)	+	+	-	ND	ND
HP-F6	CEFLQWSGKC (1)	+	+	+	ND	ND
HP-G5	CEFQQWSGKC (8)	+	+	+	-	ND
HP-D4	CETGERIVLC (1)	+	+	+	ND	ND
HP-B4	CDEKRGPNEC (1)	+	+	-	-	-
HP-B11	CVSEDIYDAC (22)	+	+	+	ND	ND
HP-D2	CHSWKPDKLC (1)	+	+	+	ND	ND

^A ^HP; Harvey Preisler.

^B ^ND not done.

The number in bracket represents the number of clones expressing the same sequence.

### Peptide binding profiles

**Figure 1:**
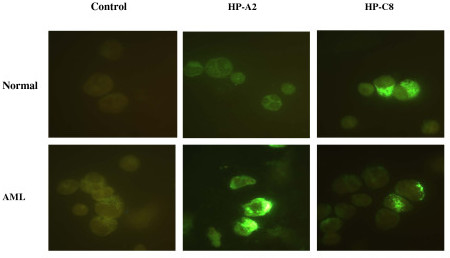
**Peptides can bind to AML cells**. AML and normal BM cells were incubated with biotinylated-peptides or non-conjugated biotin (1 μM), which were detected by streptavidin-FITC. For this specimen, HP-A2 was detected in AML but not normal cells while HP-C8 was detected in both populations.

Ten biotin-conjugated peptides were synthesized and tested for their ability to bind leukemia and normal BM cells in a cytochemistry assay (Table 2). The HP-G2 peptide differed from that of the corresponding phage in that it could bind to normal bone marrow cells. Additionally, 3 peptides showed differential binding profiles on normal bone marrow cells that differed from the phage binding of normal peripheral blood cells. This may be due to cell lineage differences in blood versus bone marrow populations. Four peptides bound to mononuclear cells from normal BM donors, HP-C8 (41%), HP-B11 (95%) HP-G2 (18%) and HP-D4 (45%), but did not bind high-density cells. This suggests that while these peptides are not specific for leukemia cells they appear to be specific for a subset of cells included in the mononuclear population. To investigate whether this subset could be CD34+ cells, three peptides HP-A2, HP-G7 and HP-B11 were tested on purified normal BM CD34+ cells. Only HP-B11 was found to bind to a small population of cells (Table 2). Importantly, the 10 peptides did not bind to human skin fibroblast cells, Hs68, which express epitopes common to multiple cell types. The 6 peptides that did not bind normal bone marrow cells together with the peptide HP-A2A (alanine substituted for arginine of HP-A2) and HP-A6 (both used in further studies) were then profiled on mononuclear cells from patients with AML (Table 3). The percentage of cells recognized by the individual peptides varied, as expected, due to the heterogeneous nature of the epitopes recognized by the peptide. Figure 1 illustrates the staining for two peptides, HP-A2 and HP-C8, on one normal and one AML specimen. HP-C8 bound cells in both specimens unlike HP-A2 that bound only AML cells. Specificity of peptide binding was further confirmed by a competition assay on cells from two different AML patients. Incubation with 100-fold concentration of non-biotinylated HP-A2 resulted in blocking the binding of the biotinylated HP-A2. Similarly, HP-G7 and HP-B6 binding was specifically blocked by the non-labeled peptide.

**Table 2:** Binding profiles of synthesized peptides on leukemia and normal bone marrow cells.

Peptide name	Peptide sequence	AML bone marrow cells	Normal bone marrow cells	Normal CD34+ cells
HP-A2	CIEETARKGC	+	-	-
HP-B6	CNHVCSRLGC	+	-	ND
HP-G7	CNELHMKQHC	+	-	-
HP-B4	CDEKRGPNEC	+	-	ND
HP-C8	CNNATVEDEC	+	+	ND
HP-G2	CNNATFEDGC	+	+	ND
HP-D4	CETGERIVLC	+	+	ND
HP-G5	CEFQQWSGKC	+	-	ND
HP-B11	CVSEDIYDAC	+	+	+
HP-D2	CHSWKPDKLC	+	-	ND

**Table 3:** Percentage of BM cells from AML patients recognized by peptides not binding to normal BM cells in a cytochemistry assay.

**Patient Number**	**HP-A2**	**HP-A2A**	**HP-G5**	**HP-G7**	**HP-D2**	**HP-B6**	**HP-B4**	**HP-A6**
15492	29	21	18	29	7	10	38	14
15480	36	ND	ND	31	ND	23	ND	ND
17026	44	ND	ND	0	0	34	0	ND
17648	32	40	31	44	50	52	19	21
16313	55	40	ND	54	48	56	25	31
16352	76	60	63	0	77	85	59	62
16278	22	30	25	0	22	41	24	22
15727	80	72	51	74	82	87	27	17
1340	32	21	34	45	46	90	31	27

**Figure 2:**
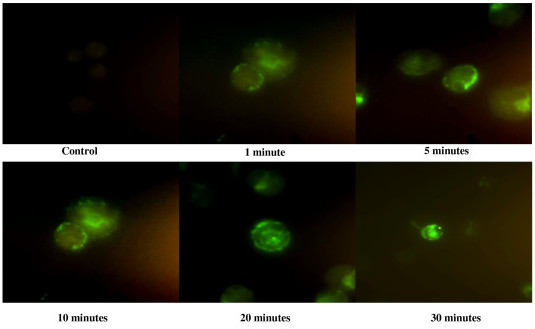
**HP-A2 is internalized after binding to AML cells**. AML cells were incubated with biotinylated-peptide HP-A2 (1 μM) at various time points. The bound peptides were initially detected at the cell surface and subsequently internalized.

Using fixed AML cells, only a cytoplasmic fluorescent signal was observed. The same binding assay was therefore repeated with fresh cells obtained from AML patients using a time-lapse format (Figure 2). The fluorescence was first observed on the surface membrane at 1 minute, followed by appearance of some signal in the cytoplasm at 5 minutes, and clear cytoplasmic signal at 30 minutes. This suggests internalization of the peptide following surface binding.

### HL60 cell differentiation induced by peptides

HL60 cells, a human promyeloid cell line, is often used as a model to study myeloid cell differentiation. All the peptides that bind AML cells were also found to bind to HL60 cells. Therefore, these cells were used to study the biological effects triggered by peptide binding. Since peptides HP-A2 and HP-G7 bind only AML cells and not normal or CD34+ bone marrow cells, they were used for these studies. HL60 cells were cultured in RPMI1640/10% FBS for 7 days with peptides HP-A2 or HP-G7 at concentrations ranging from 10^-6 ^M to 10^-4 ^M. DMSO (1.5%) was used as a positive control for differentiation [10]. As shown in Table 4, the cell proliferation was inhibited by DMSO but not by the peptides. Cells remained viable under all conditions. Differentiation was measured by following appearance of the myeloid maturation marker CD11b. Percentage of control cells expressing CD11b was of 40.6 ± 1.3% indicating a low level of spontaneous differentiation. After differentiation induced by DMSO, the CD11b marker was found on 82.2 ± 1.6% of cells. Similarly, cells incubated in the presence of 10^-4 ^M of either peptide showed increased CD11b expression with peptide HP-A2 inducing levels equal to those found with DMSO (79.7 ± 3.1%). Lower concentrations of peptide did not induce CD11b expression. Giemsa staining for morphology showed that only 5.5% of the control cells were granulocytes whereas cultures with DMSO contained 90.7% granulocytes. At 10^-4 ^M, both peptides induced a more limited morphological effect. Since the biological activity of the peptides on both marker expression and morphology was observed at only 10^-4 ^M, we limited our further studies on patient cells to this concentration. As differentiation is usually associated with arrest in the cell cycle, we looked at the correlation between CD11b expression and the cell cycle. Cultures induced to differentiate with DMSO exhibited cell cycle arrest as evidenced by an increase in G0/G1 (from 44.5 ± 9.1% to 58.0 ± 13.0%). In contrast, the peptide cultures showed no change in the proportion of cells in G0/G1 despite the marked increase in CD11b expression.

**Table 4:** Effects of DMSO, HP-A2 and HP-G7 on proliferation, cell cycle and granulocytic differentiation in HL60 cells.

	**Control**	**DMSO**		**HP-A2**			**HP-G7**	
			**10^-6 ^M**	**10^-5 ^M**	**10^-4 ^M**	**10^-6 ^M**	**10^-5 ^M**	**10^-4 ^M**

**Cell number ** **(% of the control)**	100	**38 ± 0.3**^A^	87 ± 3.7	86 ± 1.6	87 ± 1.4	95 ± 1.3	73 ± 4.7	89 ± 1.3
**G0/G1 (%)**	44.5 ± 9.1	**58.0 ± 13.0**	38.0 ± 2.3	34.0 ± 4.7	41.8 ± 6.1	43.6 ± 3.9	39.6 ± 6.0	42.5 ± 7.9
**CD11b**	40.6 ± 1.3	**82.2 ± 1.6**	38.1 ± 1.2	40.4 ± 2.7	**79.7 ± 3.1**	37.7 ± 2.9	34.1 ± 3.9	**57.8 ± 3.1**
**Granulocytic** **cells (%)**	5.5 ± 1.7	**90.7 ± 4.3**	13.0 ± 5.2	10.3 ± 3.4	**16.5 ± 4.2**	7.0 ± 1.3	10.1 ± 2.2	**15.0 ± 2.2**

^A ^All results are mean ± S.D. (n = 3).

### Peptides are not toxic to human cells

In order to show that these peptides were not directly toxic to both malignant and normal cells, viability was evaluated at 2 weeks for 2 AML and 3 normal BM specimens in the presence of a single dose of 10^-4 ^M peptide. There was no significant decrease in the viability of cells cultured in the presence of any of the 7 peptides tested and used in further studies (HP-A2, HP-A2A, HP-B4, HP-B6, HP-D2, HP-G5, HP-G7 >90% viability).

### Proliferation and Differentiation of freshly obtained human leukemia cells are altered by peptides

**Figure 3:**
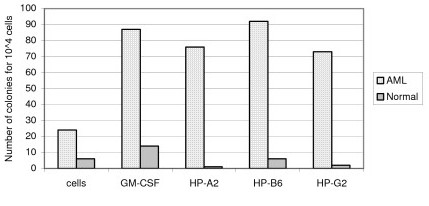
**Peptides are capable of increasing the cloning efficiency of cells from a patient with AML but not normal cells**. AML and normal cells were incubated in methylcellulose culture in the presence of peptides at 10 ^-4 ^M. After 14 days, the number of colonies was assessed.

Methylcellulose culture permits assessment of the effects of peptides on the cloning efficiency of myeloid progenitor cells. Cells were cultured with peptide for 14 days using GM-CSF as a positive control. Due to the limited number of cells available from each patient, not all peptides could be tested on all patient samples. Results from a representative patient in whom 3 different peptides were tested are presented in Figure 3. Similar studies were performed on multiple samples from different patients and are summarized in the section below. Peptides HP-A2, HP-B6 and HP-G2 induced an increase in the number of colonies in the example shown (Figure 3). This increase was equivalent to the one observed after GM-CSF stimulation. In contrast, incubation of normal cells with the peptide did not induce any change in colony number.

**Figure 4:**
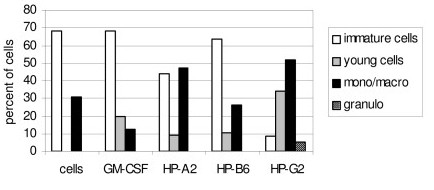
**HP-A2 and HP-G2 but not HP-B6 induced differentiation of cells from the AML patient shown in Fig.4**. AML cells were incubated in methylcellulose culture in the presence of peptides at 10 ^-4 ^M or GM-CSF (100 U/ml). After 14 days, cell morphology was evaluated. The percentage of each cell population was determined by counting 200 cells.

Cellular differentiation evaluated by morphological changes was assessed after 14 days of in vitro cultures. Using the AML specimen shown in Figure 3, the proportion of differentiated monocytes/macrophages increased after incubation with the peptides HP-A2 and HP-G2 (47% and 52% respectively). Spontaneous differentiation in control cells was only 31% (Figure 4). Peptide HP-B6 failed to induce differentiation in this patient. The proportion of mature monocytes/macrophages was significantly higher in the presence of peptides than in the presence of GM-CSF.

### Summary of the effects of 7 different peptides on proliferation and differentiation of AML progenitor cells

**Figure 5:**
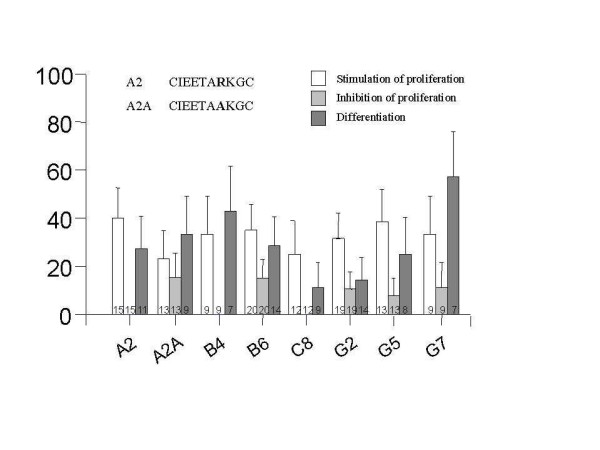
**The probability of a biologic response of AML patients to each peptide**. Cells from different AML specimens were incubated in the presence of peptides at 10 ^-4 ^M for 14 days in methylcellulose culture. The numbers of colonies and cell morphology were evaluated for each specimen. The probability of the AML specimen to respond to the peptide (increase of colony number, decrease of colony number and cell differentiation) was assessed. The numbers on the bars indicate the number of patients evaluated.

The biologic effects of 7 different peptides on the behavior of AML progenitor cells from 20 patients were evaluated. Five peptides do not bind to normal bone marrow cells (HP-A2, HP-B4, HP-B6, HP-G5 and HP-G7) while 2 peptides bind to both leukemia and normal cells (HP-C8 and HP-G2). It was of interest to include both types of peptides to see whether there was an obvious difference. Figure 5 is a summary of the biological activity of each peptide. Table 5 is a summary of clinical characteristics and biological activity induced by at least one peptide for each individual patient. As seen in Figure 5 for example, 40% of 15 AML patients incubated with HP-A2 showed induced proliferation. Eleven of these 15 patients were evaluated for differentiation and 25% showed a response.

**Table 5:** Clinical characteristics of AML patients at the time the bone marrow was obtained and biological response after 14 days of culture in the presence of GM-CSF (100 U/ml) or one peptide (10^-4 ^M).

Patient number	Age	FAB	Blast Count	Karyotype Abnormalities	Prior MDS	Prior Toxic Exposure	Increase in colony number		Decrease in colony number		Differentiation	
							peptide	GM-CSF	peptide	GM-CSF	peptide	GM-CSF

15857	76	M0	20	yes	yes		-	-	+^A^	-	+	+
14742	58	M0	88	no	no	yes	-	-	-	-	ND	ND
14924	76	M1	8		yes		+	+	-	-	-	-
16249		M1	77				+	-	-	-	-	-
14372	56	M1	49	no	yes	yes	+	+	-	-	-	-
14122	72	M1	63	yes	yes	yes	+	+	-	-	ND	ND
15492	45	M1	55	no	no	yes	-	+	+	-	+	-
15480	55	M1	88		no	yes	-	ND	-	ND	-	ND
14373	66	M1	69	yes	yes		+	+	+	-	+	+
15071	66	M1	27	no	yes		-	-	-	-	-	-
15019	77	M2	34	no	yes	no	+	+	-	-	+	+
15891	58	M2	52	yes			-	+	-	-	+	+
16313	48	M2	38	no	no	no	+	-	-	-	+	+
11369	33	M2	20	yes	yes	yes	-	-	-	-	ND	ND
14996	48	M3		yes	no	no	-	-	-	-	ND	ND
16352	74	M4	34	yes	yes	no	+	-	+	-	+	-
16278	25	M4	35		no	no	+	+	-	-	+	-
14499	74	Unclas-sified	3			no	-	+	+	-	+	+
13459	59	Un-known	80		no	yes	+	+	-	-	-	-

^A ^A modification of cell behavior in response to GM-CSF or to at least one peptide is noted +

All peptides induced proliferation in at least several patient samples. Four peptides however could also inhibit colony formation in a percentage of the samples (18% with peptide HP-B6, 10% with peptides HP-G2 and HP-G7, 8% with peptide HP-G5). Peptides HP-C8 and HP-G2 were the least (10–20% of marrow specimens studied) and peptides HP-B4 and HP-G7 were the most likely to induce differentiation (45% and 60% respectively). Peptide induced differentiation ranged between 9–75% and spontaneous differentiation ranged between 5.5–29%.

As seen in Table 5, an increase in colony number (proliferation) was induced in 10/19 specimens by at least one peptide (column 8). Differentiation (column 12) was seen in 9/15 for at least one peptide. Three specimens showed both differentiation and proliferation after culture with a specific peptide. Interestingly, in only 2 AML specimens, inhibition of proliferation by a specific peptide was accompanied by cell differentiation (AML #15857 and #14499).

### Single amino acid mutation effects biological activity

Binding specificity should be dependent on peptide conformation. Mutation of one of the amino acids may alter the binding properties and/or biological activity. An internal arginine residue (large and polar) of peptide HP-A2 (IEETA**R**KG) was replaced by an alanine residue (small and non-polar); HP-A2A. Two additional peptides contained a polar amino acid at the same position HP-G7 and HP-B6 (NELHM**K**QH, and NHVCS**R**LG). These three peptides did not bind normal BM cells suggesting that this amino acid may be important to peptide specificity. While binding properties of the mutated peptide were not changed (Table 3), the number of patients in whom proliferation was stimulated was significantly lower (from 40 to 22%). In addition, this peptide actually inhibited proliferation in a number of samples (16%) (Figure 5).

## Discussion

Therapeutic progress in AML has been painfully slow despite significant improvement in our understanding of the underlying pathology. A major conundrum in designing treatment strategies relates to the issue of targeting only the malignant cells while sparing their normal counterparts. In this work, we have shown that using a phage library, it is possible to isolate peptides that can bind and induce biologic activity only in leukemia cells. Since cell lines are often not representative of the physiological state of malignant cells, we used freshly obtained patient specimens for the initial screening, and in order to avoid isolating patient specific peptides, the screening and profiling were performed with cells from multiple patients with different myeloid leukemia subtypes. It is of interest that only 12 unique peptide sequences were found and that one sequence (HP-B11) was found in 22 clones. The redundancy of the phage sequence may be related to the copy number of each epitope expressed on hematopoietic cells. Ten synthesized peptides were re-profiled on AML and normal cells. As expected, all the peptides were able to bind to the malignant cells, but four peptides also were found to bind to normal BM cells. The peptides were able to bind to all AML-FAB subtypes in our small sample size, but the percentage of cells recognized by the peptide was lower at relapse.

We have found at least 2 peptides, HP-A2 and HP-G7, that bind only to AML cells and not to normal cells including normal CD34+ cells. Cytochemistry studies showed that after surface binding, these two peptides were internalized within minutes. Intracellular localization is a particularly attractive feature since it offers a novel method to deliver drugs and may reduce toxicity by allowing lower concentrations to be administered. In addition, these studies showed that the peptides do not always label all the leukemia cells which is not surprising since AML blasts are not a homogeneous population [11] and AML marrows frequently contain normal mononuclear cells.

In order to develop clinically useful strategies, we were looking for peptides that, upon binding, could induce some biological change in the leukemia cell growth and differentiation. The peptides that we have identified could clearly affect both these parameters although the biologic response elicited was variable. A given peptide could induce proliferation, differentiation, both or none in the cells of different AML patients. Of the two leukemia specific peptides, HP-A2 stimulated proliferation in 40% and differentiation in 25% of the patients tested while HP-G7 stimulated proliferation in 35% and differentiation in 60% of the patients. This includes some patients in whom the peptides induced proliferation followed by differentiation. To be therapeutically useful in future, the biologic effects of a given peptide would have to be defined for the individual patient. While the peptides were screened to be myeloid specific and not patient specific, the biologic activity elicited by a peptide could depend on multiple variables. The maturation state at which the AML clone is arrested is defined by the FAB subtype and this may be a factor that determines the specific response to the peptide. We have not analyzed sufficient numbers of AML patients to correlate activity with subtype. Additionally, activity could be determined by the number of epitopes on the cells of the AML clone.

Blast analysis of the sequence of the two leukemia specific peptides revealed that 7 of 8 amino acids of HP-A2 matched mostly to proteins of lower organisms. For HP-G7, the best match was 6 amino acids identical to two different proteins; the Toll-like receptor 2 and the neraminidase protein of the streptococcus pneumonia bacteria. Further studies, however, will be needed to clarify the molecular epitope that is recognized by these peptides in order to understand the mechanism(s) responsible for their biological activity.

The high concentration of peptides used for these studies is non-physiological. However, the peptide solution, administered as a single dose at the start of cell culture, was found to contain a mixture of various conformations including linear, cyclic or concatamers. It is possible that the structure exhibiting biological activity represented only a fraction of the total and its stability in the culture medium is unknown. This may explain the need for a high initial concentration to elicit a biological effect. It will be necessary in the future to identify the conformation responsible for biological activity. In addition to conformation, peptide sequence determines the nature of the biological response. For example, HP-C8 and HP-G2, identical for 6 amino acids, elicited different activities despite binding similarities. Finally, a single amino acid substitution in HP-A2 altered the ability of the peptide to induce proliferation. These observations support a specificity of the structure-function relationships of the peptide and its target.

HP-A2 and HP-G7 were also found to bind HL60 cells that have often been used as a model to study myeloid cell growth and differentiation. We have used HL60 cells to study the effect of these two peptides on cell cycle and differentiation. While DMSO was found to increase CD11b expression and arrest the cell cycle, we found that CD11b expression was increased by the peptides without concomitant cell cycle arrest. Studies with other differentiating agents show accumulation of cells during differentiation in G1 by day 3 or day 4 [12-14], we have not found a G1 block or decrease in cell number in peptide treated differentiating cells even after 7 days of culture. Thus the mechanism of differentiation by the peptides appears to be novel and different than that of the known conventional differentiating agents. It may be that the binding of these peptides results in an uncoupling of the expression of the proliferation and differentiation markers. In the absence of growth arrest, it is conceivable that the peptides can also be used concomitantly with chemotherapeutic agents which depend upon cellular synthesis to increase the therapeutic index.

The leukemia specific peptides are expected to be non-toxic based on in vitro results, and theoretical in vivo clinical applications are multiple. Coupled to toxins, the peptides could specifically target and kill tumor cells as first line therapy and/or as therapy targeting residual disease. When coupled with an imaging reagent, the peptides could be used to locate sites of tumor sanctuary. The peptides could also be combined with peptides that selectively target the tumor vasculature [15]. The challenge now is to identify the molecular target on the surface of leukemia cells that binds these peptides, demonstrate that the in vitro effects are reproducible in vivo by conducting similar experiments in animal models and finally translating these findings into clinical trials for human use; areas that are currently being actively pursued in our laboratory at present.

## Competing interests

The authors declare that they have no competing interests.

## Authors' contributions

NG participated in study design, conducted functional studies, analysis and wrote the manuscript. ED participated in study design, isolated the peptides conducted functional studies and analysis. AR participated in study concept, design and coordination and wrote the manuscript. All authors read and approved the manuscript.
